# A systematic review of a novel alloplast carbonate apatite granules

**DOI:** 10.3389/fdmed.2024.1418039

**Published:** 2024-08-16

**Authors:** Martha E. Nunn, Courtney Rudick, Masahiko Nikaido, Takanari Miyamoto

**Affiliations:** ^1^ Nunn Biostatistical Solutions, Omaha, NE, United States; ^2^ Private Practitioner, Omaha, NE, United States; ^3^ Tokyo Dental College, Tokyo, Japan; ^4^ Private Practitioner, Tokyo, Japan; ^5^ Department of Periodontics, Creighton University School of Dentistry, Omaha, NE, United States

**Keywords:** alloplast, hard-tissue graft, carbonate apatite granules, cytrans granules, artificial bone

## Abstract

The objectives of this study are to provide a systematic review of a novel alloplastic hard-tissue grafting material, carbonate apatite granules (CO3Ap-granules), to provide a clinical case presentation of CO3Ap-granules in periodontal surgery. The following three electronic databases were searched independently by two of the authors (MN) and (CR): National Library of Medicine [MEDLINE (PubMed) and ClinicalTrials.gov], EMBASE (OVID) and the Cochrane Central Register of Controlled Trials (CENTRAL). After searching electronic databases, select journals in periodontics and implantology were also manually searched. Of the 43 studies identified from the systematic review, the following classifications were determined: (1) *in vitro* studies – 5 studies, (2) animal studies – 28 studies, (3) clinical studies – 7 studies, (4) reviews – 3 studies. Results from selected animal studies and all human studies were summarized. These results demonstrate that the novel alloplast CO3Ap-granules has the potential ability to stimulate new bone formation while CO3Ap-granules simultaneously resorb over time. Replacement of CO3Ap-granules with new bone formation has been shown to be comparable to autogenous bone grafting with one study showing superior results to a bovine-derived xenograft.

## 1 Introduction

Hard-tissue grafting is integral to the practice of periodontics, including repair of alveolar bone (1), periodontal pocket elimination, gain in periodontal attachment, sinus augmentations, alveolar ridge preservation (2, 3), alveolar ridge augmentation (4), an adjunct to implant placement (5), to facilitate socket healing with placement of immediate implants (5, 6), and in treatment of peri-implantitis (7). Different types of hard-tissue grafts include autografts, allografts, xenografts, and alloplasts (6, 8, 9). Autografts are defined as tissue moved from one location to another site within the same species. Allografts are defined as tissue moved from one person to another genetically dissimilar person, such as freeze-dried bone allografts (FDBA) or demineralized freeze-dried bone (DFDBA). While allografts are widely used in the field of periodontics, risks from allografts include antigenicity and risk of infection (8), although freeze-drying markedly reduces these risks (10, 11). Xenografts are defined as tissue transferred from one species to another species, such as bovine-derived hard-tissue graft. The deproteinized bovine bone matrix (DBBM) is mainly xenograft materials used in alveolar bone defects and in sinus floor augmentation (included citations). DBBM consists of hydroxyapatite prepared by alkaline and heat treatment for eliminating the organics of the bone. However, bovine-derived graft biomaterials may theoretically carry a risk of prion/bovine spongiform encephalopathy transmission to patients (6, 12).

In contrast to autografts, allografts, and xenografts, an alloplast is completely artificial. Hence, alloplasts are defined as artificial tissue, and since alloplasts are not dependent on tissues derived from living beings, such as allografts from other humans or xenografts from animals, the availability of alloplasts is virtually unlimited since the single limiting factor is the least available material used to fabricate an alloplast. Alloplastic graft is usually made from hydroxyapatite, which is a natural mineral component of bone. Thus, they have no risk for cross infection or disease transmission, which might be a possibility with the use of allografts and xenografts (6, 8, 9). If an alloplast has similar properties to an autograft, such as osteoconductivity and osteoinductivity, that alloplast would be the perfect solution for bone grafting. Early bone substitutes included hydroxyapatite [HAp: Ca10(PO4)6-(OH)2], β-tricalcium phosphate [β-TCP: Ca3(PO4)2], biphasic calcium phosphate, BCP; a mixture of HAp and β-TCP, calcium sulfate [CaSO4·2H2O], and bioglass (13–15).

CO3Ap-granules are a novel alloplastic material that are capable of inducing new bone generation, while also resorbing from the graft site, leaving an area of newly formed bone and blood vessels, without any remaining graft material (15–17). One of the unique ways CO3Ap-granules accomplish this is through the activation of both osteoclasts and osteoblasts from osteoprogenitor cells within the graft site (18). CO3Ap-granules induce the transcription of osteoclast differentiation markers such as cathepsin K and tartrate-resistant acid phosphatase allowing osteoclasts to develop in direct contact with the CO3Ap-granules without any intermediary layers of fibrous tissue (19). Both the carbonate apatite and newly differentiated osteoclasts in turn activate osteoblast differentiation via upregulation of alkaline phosphatase, osteopontin, and osteocalcin (16–18, 20). Osteoblasts near CO3Ap-granules lay down new type I collagen, the most abundant bone matrix protein expressed in both mature bone and as the main organic component of immature bone, indicating the osteoinductive capabilities of the granules (17, 18). The presence of osteocalcin and osteopontin found in the newly formed bone around CO3Ap-granules indicates extracellular matrix synthesis and bone matrix mineralization necessary for strong replacement bone (18). The CO3Ap-granules act as a scaffold for both osteogenesis and angiogenesis to promote vascularization while allowing autogenous bone to grow adjacent to, and eventually replace, the CO3Ap-granules (17, 20). In addition to inducing osteoclast differentiation, CO3Ap-granules release certain ions in aqueous solutions, namely carbonic acid, phosphoric acid, and calcium, which reduce the pH of the immediate area promoting osteoclast activity. Because CO3Ap-granules are stable at physiologic pH, but dissolve at low pH, the release of ions allows both bone remodeling and granule dissolution to occur at the same time (16, 17, 20).

A successful hard-tissue graft involves applying the hard-tissue grafting material, a period of healing, the hard-tissue grafting material becoming incorporated into the bone, revascularization, and finally, assuming the form desired by the clinician (6). Early hard-tissue grafts were viewed as lattice structures with the success of these bone-like lattice structures judged primarily by the mechanical stress that could be applied to the area containing a hard-tissue graft (16, 17, 20). Modern hard-tissue grafts are treated as biological structures. Mechanical stress, shear stress, contouring, and regeneration of new bone in the remodeling stage are all part of the long-term healing of modern hard-tissue grafts (8).

The purpose of this paper is to provide a systematic review of a novel alloplast, CO3Ap-granules (Cytrans Granules®: GC America, Alsip, IL), and to demonstrate a clinical case with long term outcome utilizing carbonate apatite granules in periodontal regeneration. For background, a brief overview of the different types of hard-tissue grafts used in periodontics and implantology is provided as Supplementary Material.

## 2 Materials and methods

This systematic review was conducted in accordance with the Preferred Reporting Items for Systematic Reviews and Meta-Analysis (PRISMA) and Cochrane guidelines. Supplementary Diagram 1 shows the PRISMA flowchart and PRISMA checklist. The following focus question is based on population, intervention, comparison, and outcome (PICO) as shown in Table 1.

**Table 1:** PICO study design.

Component	Description
Population (P)	Subjects in need of a bone graft
Intervention (I)	Carbonate apatite granules (Cytrans®)
Comparison (C)	Other grafting material
Outcome (O)	Varies by study
Study Design (S)	Animal studies and clinical studies (divided into separate sections for clarity)
Focus Question	What is the effect of using carbonate apatite granules (Cytrans®) for oral bone grafts in subjects, when compared to either unassisted healing or other bone grafting material, such as bovine-derived xenograft (Bio-Oss®)?

What is the effect of CO3Ap-granules intraoral graft when compared to unassisted healing or other intraoral bone graft materials?

An initial electronic systematic search was performed independently by two of the authors (MN) and (CR). The following electronic databases were searched: National Library of Medicine [MEDLINE (PubMed) and ClinicalTrials.gov], EMBASE (OVID) and the Cochrane Central Register of Controlled Trials (CENTRAL).

The following search strategy was designed for the MEDLINE (PubMed) database and then modified accordingly for other database engines: (“carbonate apatite” or “carbonated apatite” or “Cytrans”) and (“periodontal surgery” or “periodontology” or “implant” or “oral surgery”). The most recent electronic database search was conducted on November 1, 2023. In addition to the electronic search, further manual search of the following journals from 2012 to November 1, 2023 was also conducted: *Journal of Periodontology*, *Journal of Clinical Periodontology*, *Clinical Oral Implants Research*, *Journal of Dental Research*, *International Journal of Oral and Maxillofacial Implants*, *International Journal of Oral and Maxillofacial Surgery*, and *International Journal of Periodontics and Restorative Dentistry*.

Following the initial systematic search, all the titles and abstracts were scanned independently by two investigators (MN and CR), followed by the full-text assessment of the potentially eligible studies. In case of any disagreement regarding study selection, a third investigator (TM) was contacted. Studies unrelated to using CO3Ap-granules as a hard-tissue grafting material were eliminated.

## 3 Results

### 3.1 Types of publications

Publications we encountered in our systematic review are classified into four categories: (1) *in vitro* studies (5 studies), (2) animal studies (28 studies), (3) human clinical studies (7 studies), and (4) review articles (3 studies). For the purposes of this systematic review, we will focus on the second and third categories with particular attention to human clinical studies. Tables of all studies involving CO3Ap-granules, used as alloplast grafting material, are shown in Table 2 (*in vitro* studies), Table 3 (animal studies), Table 4 (human clinical studies), and Table 5 (review articles). Normally, systematic reviews are restricted to human clinical studies. However, because of the large number of animal studies and a very limited number of human clinical studies, all animal studies and all human clinical studies are presented in Tables 3, 4. A subset of the most significant animal studies (10 of 28 animal studies) and all human clinical studies are presented in the results.

**Table 2:** *In vitro* studies.

Authors	Year	Title	*In vitro* results
Ishikawa K, Matsuya S, Lin X, et al.	2010	Fabrication of low crystalline B-type carbonate apatite block from low crystalline calcite block	Low crystalline B-type carbonate apatite (CO3Ap) block was fabricated by compositional transformation based on dissolution precipitation reaction using microporous low crystalline calcite block as a precursor
Nagai H, Kobayashi-Fujioka M, et al.	2015	Effects of low crystalline carbonate apatite on proliferation and osteoblastic differentiation of human bone marrow cells	The number of cells on HAp was higher than that on CO3Ap until day 7, after which the number of cells was similar. hBMSC proliferated more significantly on tissue culture plate than on HAp and CO3Ap discs. In contrast, hBMCs incubated on CO3Ap demonstrated much higher expression of osteoblastic markers of differentiation, such as type I collagen, alkaline phosphatase, osteopontin and osteocalcin, than hBMCs on HAp.
Tsuchiya A, Freitas PP, Nagashima N, Ishikawa K.	2021	Influence of pH and ion components in the liquid phase on the setting reaction of carbonate apatite granules	The presence of Ca2+ in acidic phosphate solution is key for preventing CO3Ap washout
Fujioka-Kobayashi M, Katagiri H, et al.	2021	The impact of the size of bone substitute granules on macrophage and osteoblast behaviors *in vitro*	Bone substitute (BS) size might influence the clinical outcomes of guided bone regeneration (GBR) procedures. Gene expression experiments in M*φ*s revealed few differences between the two sizes of each BS, although higher CD206 mRNA levels were observed in the CO3Ap-M group than in the respective S-size groups on day 1.
Fujioka-Kobayashi M, Miyamoto Y, et al.	2022	Osteoclast behaviors on the surface of deproteinized bovine bone mineral and carbonate apatite substitutes *in vitro*	limited results showed that CO3Ap provided a favorable surface for osteoclast differentiation, as well as osteoblasts, compared to deproteinized bovine bone mineral *in vitro*

**Table 3:** Animal studies.

Authors	Year	Title	Species	Study results
Ayukawa Y, Suzuki Y, Tsuru K, et al.	2015	Histological comparison in rats between carbonate apatite fabricated from gypsum and sintered hydroxyapatite on bone remodeling	Rat – tibia	CO3Ap granules mimicked natural bone remodeling at 2 and 4-weeks with osteoblast bone matrix apposition and osteoclast resorption of the granules. In the same timeframe hydroxyapatite granules did not form new bone but were sometimes encapsulated by fibrous tissue instead.
Rakhmatia YD, Ayukawa Y, Furuhashi A, Koyano K.	2018	Carbonate apatite containing statin enhances bone formation in healing incisal extraction sockets in rats	Rat – jaw	CO3Ap containing statin, followed by CO3Ap alone, showed the most bone volume, trabecular thickness, and trabecular separation compared the hydroxyapatite with or without statins.
Zhang X, Atsuta I, Narimatsu I, et al.	2021	Replacement process of carbonate apatite by alveolar bone in a rat extraction socket	Rat – jaw	CO3Ap is absorbed gradually by osteoclasts in extraction socket very similar to normal physiologic remodeling conditions allowing room for new bone formation in the area of extractions.
Egashira Y, Atsuta I, Narimatsu I, et al.	2022	Effect of carbonate apatite as a bone substitute on oral mucosal healing in a rat extraction socket: *in vitro* and *in vivo* analyses using carbonate apatite	Rat – jaw	At two weeks post-implantation, soft tissues healed around CO3Ap and connective tissue was restored around the site prompted by high collagen expression by fibroblasts adjacent to the CO3Ap material.
Atsuta I, Mizokami T, Jinno Y, et al.	2022	Synergistic effect of carbonate apatite and autogenous bone (AB) on osteogenesis	Rat – tibia	A mixture of CO3Ap and AB combines the osteogenic and osteoinductive properties of each component, which may provide bone formation over a wide area
Takahashi R, Atsuta I, Narimatsu I, et al.	2023	Evaluation of carbonate apatite as a bone substitute in rat extraction sockets from the perspective of mesenchymal stem cells	Rat – jaw	CO3Ap-filled extraction sockets accumulated mesenchymal stem cells (MSCs), and MSCs cultured in the presence of CO3Ap produced large amounts of growth factors.
Matsuura A, Kubo T, Doi K, et al.	2009	Bone formation ability of carbonate apatite-collagen scaffolds with different carbonate contents	Rabbit – femur	CO3Ap scaffolds in rabbit femur implants were completely indistinguishable from surrounding bone at 24 weeks.
Ishikawa K, Arifta TI, Hayashi K, Tsuru K.	2018	Fabrication and evaluation of interconnected porous carbonate apatite from alpha tricalcium phosphate spheres	Rabbit – tibia	Porous CO3Ap shows greater bone formation in tibial defects than dense CO3Ap at 4 and 12 weeks.
Fujioka-Kobayashi M, Tsuru K, Nagai H, et al.	2018	Fabrication and evaluation of carbonate apatite-coated calcium carbonate bone substitutes for bone tissue engineering	Rabbit – femur	Despite calcium carbonate showing increased calcium ion release and cell proliferation *in vitro*, *in vivo* CO3Ap showed superior osteoconductive properties at 8-weeks.
Fujisawa K, Akita K, Fukuda N, et al.	2018	Compositional and histological comparison of carbonate apatite fabricated by dissolution–precipitation reaction and Bio-Oss	Rabbit – femur	CO3Ap granules prompted a significantly larger amount of new cortical bone formation compared to Bio-Oss® at 4-weeks and were partially replaced by the new bone at 8 weeks, whereas Bio-Oss® remained in the area unchanged.
Ishikawa K, Munar ML, Tsuru K, Miyamoto Y.	2019	Fabrication of carbonate apatite honeycomb and its tissue response	Rabbit – femur	New bone completely penetrated the CO3Ap honeycomb blocks with osteoclast and osteoblast activity on the newly formed osteocytes. Ongoing bone formation also included new blood vessel networks within the area.
Sakemi Y, Hayashi K, Tsuchiya A, et al.	2019	Fabrication and histological evaluation of porous carbonate apatite block from gypsum block containing spherical phenol resin as a porogen	Rabbit – femur	Macroporous CO3Ap formed more bone faster than non-macroporous CO3Ap or macroporous Hap. Complete bone reconstruction was achieved at 12 weeks for the macroporous CO3Ap
Hayashi K, Kishida R, Tsuchiya A, Ishikawa K.	2019	Honeycomb blocks composed of carbonate apatite, β-tricalcium phosphate, and hydroxyapatite for bone regeneration: effects of composition on biological responses	Rabbit – femur	CO3Ap demonstrated faster bone formation than β-TCP and hydroxyapatite materials at 4 and 12-weeks. CO3Ap was resorbed only by osteoclast activity, whereas hydroxyapatite did not dissolve and β-TCP dissolved rapidly without
Tanaka K, Tsuchiya A, Ogino Y, et al.	2020	Fabrication and histological evaluation of a fully interconnected porous CO_3_Ap block formed by hydrate expansion of CaO granules	Rabbit – femur	CO3Ap fabricated by a dissolution−precipitation reaction from a precursor exhibits excellent osteoconductivity and is readily replaced by bone.
Hayashi K, Kishida R, Tsuchiya A, Ishikawa K.	2020	Granular honeycombs composed of carbonate apatite, hydroxyapatite, and β-tricalcium phosphate as bone graft substitutes: effects of composition on bone formation and maturation	Rabbit – femur	CO3Ap implant groups showed new mature bone formation by 4-weeks after grafting with a large portion of the CO3Ap replaced by new bone at week 12. β-TCP grafting sites showed some areas where new bone was not formed even after dissolution of the grafting material and still showed immature bone present at 12-weeks.
Akita K, Fukuda N, Kamada K, et al.	2020	Fabrication of porous carbonate apatite granules using microfiber and its histological evaluations in rabbit calvarial bone defects	Rabbit - calvarial bone	Pore size has an impact on new bone formation. CO3Ap blocks with larger pore sizes showed increased mature bone formation at 4 and 8-weeks although the results did not reach the threshold for statistical significance.
Deguchi K, Nomura S, Tsuchiya A, et al.	2021	Effects of the carbonate content in carbonate apatite on bone replacement	Rabbit – femur	The rate of granule resorption and replacement by newly formed bone increases as carbonate content increases from 0.9–8.3%.
Elsheikh M, Kishida R, Hayashi K, et al.	2022	Effects of pore interconnectivity on bone regeneration in carbonate apatite blocks	Rabbit – femur	CO3Ap with larger interconnected pore volumes lead to more new bone formation throughout the entire implant site. At 12-weeks the border between original host-bone and CO3Ap new bone formation was indistinguishable.
Kudoh K, Fukuda N, Akita K, et al.	2022	Reconstruction of rabbit mandibular bone defects using carbonate apatite honeycomb blocks with an interconnected porous structure	Rabbit – jaw	The morphology of CO3Ap honeycombs (HCBs) promotes bone infiltration from both lateral ends, and it inhibits the invasion of soft tissue from the lateral sides. CO3Ap HCBs showed an excellent tissue response and good osteoconductivity. It may be useful in the reconstruction of large mandibular bone defects.
Kim DK, Lee SJ, Cho TH, et al.	2010	Comparison of a synthetic bone substitute composed of carbonated apatite with an anorganic bovine xenograft in particulate forms in a canine maxillary augmentation model	Beagle dogs – mandible	Porous structures have a great impact on the formation of new bone in osteoconductive implant materials.
Ishikawa K, Miyamoto Y, Tsuchiya A, et al.	2018	Physical and histological comparison of hydroxyapatite, carbonate apatite, and β-tricalcium phosphate bone substitutes	Dogs – mandible	Cytrans® Granules are stable under normal physiological conditions but are resorbed in conditions that mimic Howship's lacunae. Cytrans® Granules showed new bone formation throughout the entire defect area by 4-weeks and bone increased through at least week 12 when Cytrans® Granules were resorbed. Cerasorb® showed limited bone at 4-weeks, but had bone formation through week 12.
Mano T, Akita K, Fukuda N, et al.	2019	Histological comparison of three apatitic bone substitutes with different carbonate contents in alveolar bone defects in a beagle mandible with simultaneous implant installation	Beagle dogs – mandible	Cytrans® Granules showed 75% new bone formation 12 weeks after surgery and were able to restore alveolar ridge contour whereas the alveolar ridge was not sufficient when using NEOBONE® or Bio-Oss® grafting materials.
Sato N, Handa K, Venkataiah VS, et al.	2020	Comparison of the vertical bone defect healing abilities of carbonate apatite, β-tricalcium phosphate, hydroxyapatite and bovine-derived heterogeneous bone	Beagle dogs – mandible	Cytrans® Granules compared to Cerasorb®, Neobone®, and Bio-Oss® in a three-wall bone defect: all four showed similar healing in cementum and periodontal ligaments; however, Cytrans showed the fastest bone healing with osteoclast and endothelial cell recruitment at 4 weeks, compared to 8 weeks in the other groups.
Shirakata Y, Setoguchi F, Sena K, et al.	2022	Comparison of periodontal wound healing/regeneration by recombinant human fibroblast growth factor-2 combined with β-tricalcium phosphate, carbonate apatite, or deproteinized bovine bone mineral in a canine one-wall intra-bony defect model	Beagle dogs – mandible	CO3Ap treatment group showed more recombinant human fibroblast growth factor stability than β-TCP or DBBM. DBBM with rhFGF-2 showed the highest amount of periodontal soft tissue regeneration.
Suehiro F, Komabashiri N, Masuzaki T, et al.	2022	Efficacy of bone grafting materials in preserving the alveolar ridge in a canine model	Beagle dogs – mandible	Implant placement in regenerated bone with β-TCP, bovine bone substitue, or CO3Ap. All groups showed bone healing and similarities in insertion torque, osseointegration, and implant stability. Bone levels were significantly reduced in the bovine bone substitute group at 5 weeks post-implantation.
Fukuda N, Ishikawa K, Miyamoto Y.	2022	Alveolar ridge preservation in beagle dogs using carbonate apatite bone substitute	Beagle dogs – mandible	CO3Ap granules showed 1.0 mm higher alveolar bone height and 2.0 mm buccal alveolar bone restoration at 4-weeks and 9-weeks compared to control. CO3Ap granules demonstrated complete continuity of bone formation from buccal to palatal side of alveolar bone defect by 4 weeks post surgery.
Nagayasu-Tanaka T, Anzai J, et al.	2023	Effects of combined application of fibroblast growth factor (FGF)-2 and carbonate apatite for tissue regeneration in a beagle dog model of one-wall periodontal defect	Beagle dogs – mandible	FGF-2 with CO3Ap enhances new bone formation and maintains existing bone adjacent to defect, which suggests that FGF-2 with CO3Ap could promote periodontal regeneration in severe bony defects of periodontitis patient.
Takeuchi S, Fukuba S, Okada M, et al.	2023	Preclinical evaluation of the effect of periodontal regeneration by carbonate apatite in a canine one-wall intrabony defect model	Beagle dogs – mandible	This study showed the safety and efficacy of CO3Ap for periodontal regeneration in one-wall intrabony defects in dogs. CO3Ap has a better ability to integrate with bone than β-TCP.

**Table 4:** Clinical studies.

Authors	Year	Title	Clinical study parameters
Kudoh K, Fukuda N, Kasugai S, et al.	2018	Maxillary sinus floor augmentation using low-crystalline carbonate apatite granules with simultaneous implant installation: first-in-human clinical trial	Sinus floor augmentation, 8 patients, 12 month study
Nakagawa T, Kudoh K, Fukuda N, et al.	2019	Application of low-crystalline carbonate apatite granules in 2-stage sinus floor augmentation: a prospective clinical trial and histomorphometric evaluation	Sinus floor augmentation, 13 patients, 18 month study
Ogino Y, Ayukawa Y, Tachikawa N, et al.	2021	Staged sinus floor elevation using novel low-crystalline carbonate apatite granules: prospective results after 3-year functional loading	Sinus floor elevation, 13 patients, 3 year study
Nagata K, Fuchigami K, Kitami R, et al.	2021	Comparison of the performances of low crystalline carbonate apatite and Bio-Oss in sinus augmentation using three dimensional image analysis	Bio-Oss® vs Cytrans® Granules in sinus lift, 6 month study, *Favorable to Cytrans
Kitamura M, Yamashita M, Miki K, et al.	2022	An exploratory clinical trial to evaluate the safety and efficacy of combination therapy of REGROTH and Cytrans granules for severe periodontitis with intrabony defects	Flap operation, 10 patients, Regroth with Cytrans® Granules supplementation, 36 week study
Fukuba S, Okada M, Iwata T.	2023	Clinical outcomes of periodontal regenerative therapy with carbonate apatite granules for treatments of intrabony defects, Class II and Class III furcation involvements: A 9-month prospective pilot clinical study	Periodontal regenerative therapy with CO3Ap granules for intrabony defects and Class II furcation involvement appear to be clinically safe and effective. 7 teeth in 4 patients.
Funato A, Katayama A, Moroi H.	2023	Novel synthetic carbonate apatite as bone substitute in implant treatments: case reports	This paper presents 3 cases of immediate implant placement, lateral GBR and vertical GBR. CO3Ap was utilized with new bone produced as CO3Ap is resorbed

**Table 5:** Review articles.

Authors	Year	Journal	Title
Ishikawa, K.	2019	Journal of the Ceramic Society of Japan	Carbonate apatite bone replacement: learn from the bone
Ishikawa, K.	2010	Materials	Bone substitute fabrication based on dissolution-precipitation reactions
Ishikawa K, Hayashi K.	2021	Science and Technology of Advanced Materials	Carbonate apatite artificial bone

### 3.2 Significant animal studies (10 of 28 animal studies presented)

In a study comparing CO3Ap-granules to sintered hydroxyapatite in defects created in the tibia of rats, it was found that CO3Ap-granules more closely mimicked the bone matrix with direct apposition of new bone via osteoblasts and osteoclastic resorption with new bone replacing CO3Ap-granules (21). In another study that compared the healing sockets of mandibular incisors in rats where CO3Ap-granules with and without statin were compared to hydroxyapatite (Neobone®, CoorsTek, Tokyo, Japan), with and without statin and an untreated control, CO3Ap-granules combined with statins produced the greatest bone mineral density, which suggests that CO3Ap-granules with statins may promote bone healing in the socket and may be applicable to alveolar bone preservation following tooth extraction (22). In another study of the molar sockets of rats, CO3Ap-granules was compared to autogenous bone and control. After 5 days, osteoclasts were observed near the CO3Ap-granules and the bone thickness observed for the CO3Ap-granules bone sockets was similar to autogenous bone sockets. And as observed from previous rat studies, new bone completely replaced CO3Ap-granules (17). In another study of the soft-tissue healing of molar sockets of rats where either hydroxyapatite or carbonate apatite was placed in the sockets, carbonate apatite was found to promote soft-tissue healing by accelerating wound closure with connective tissue compared to hydroxyapatite (23). In a 2023 study on the healing of rat extraction sockets, researchers focused on mesenchymal stem cell activity, which is thought to have a key role in wound healing. In this study of rat extraction sockets, they compared CO3Ap-granules, hydroxyapatite, and β-tricalcium phosphate (Cerasorb®, Curasan Inc., Frankfurt, Germany), with CO3Ap-granules producing overall greater mesenchymal stem cell activity, which suggests that CO3Ap-granules promote wound healing in the oral cavity (24).

In a study comparing CO3Ap-granules to a bovine-derived xenograft in rabbit femur defects, at 4 weeks, CO3Ap-granules outperformed the bovine-derived xenograft with a significantly larger amount of new bone deposited while CO3Ap-granules spontaneously resorbed as it was replaced by new bone. At 8 weeks, bovine-derived granules showed no change in size compared to CO3Ap-granules that gradually decreased in size because they were replaced with bone. However, the difference in new bone formation between CO3Ap-granules and bovine-derived granules at 8 weeks was not statistically significant (25). In a 2019 study of rabbit femur defects by Hayashi, et al., CO3Ap-granules, hydroxyapatite, and β-tricalcium phosphate were compared. Again, CO3Ap-granules outperformed both hydroxyapatite and β-tricalcium phosphate with CO3Ap-granules having 14.3-fold and 4.3-fold greater mature bone at 4 weeks compared to hydroxyapatite and β-tricalcium phosphate, respectively, and 7.5-fold and 1.4-fold higher mature bone at 12 weeks. In addition, CO3Ap-granules readily resorbed and were replaced by new bone while hydroxyapatite never resorbed (26). In a 2019 study by Ishikawa, et al., where a rabbit femur defect was filled with carbonate apatite honeycomb blocks, new bone completely penetrated the carbonate apatite honeycomb blocks. Osteoclasts and osteoblasts were also found on the newly formed bone (27).

In a hybrid dog mandible defect model, a comparison was made among CO3Ap-granules, hydroxyapatite, and β-tricalcium phosphate hard-tissue grafts. As in the rabbit model, CO3Ap-granules demonstrated the highest level of bone formation (28). A comparison of healing of vertical bone defects in beagle dogs was conducted to compare CO3Ap-granules, bovine-derived xenograft, β-tricalcium phosphate, hydroxyapatite, and a sham control. At 4 weeks, CO3Ap-granules demonstrated significantly greater bone formation than either the sham control group or the bovine-derived xenograft group. CO3Ap-granules also tended to have greater bone formation than either β-tricalcium phosphate or hydroxyapatite, although these differences did not achieve statistical significance. This study indicates that CO3Ap-granules initially promoted rapid bone formation, and hence, CO3Ap-granules may be a reliable bone substitute in the treatment of vertical bone defects as the result of periodontitis (29).

### 3.3 Human clinical studies

In the first human clinical trial in 2019, two sizes of CO3Ap-granules were used in 8 subjects for a sinus floor augmentation while simultaneously placing implants. The initial mean maxillary molar bone height was 5.2 ± 0.8 mm preoperatively with the post-augmentation height being 14.0 ± 1.9 mm. At 12 months, the mean maxillary bone height was 11.7 ± 0.6 mm so that total resorption after a year was 16.4%, which compares favorably with historical controls of autogenous bone augmentation and autogenous bone, bovine-derived xenograft mixture augmentation with resorption of 23.1% and 18.9%, respectively (30). This first clinical trial suggests that CO3Ap-granules is a safe, useful bone substitute for sinus floor augmentation (20).

In a two-stage sinus floor augmentation clinical study, 13 subjects with 17 implants were enrolled. The mean residual bone height was 3.5 ± 1.3 mm with a mean post-augmentation height of 13.3 ± 1.7 mm achieved with CO3Ap-granules. Mean bone height was found to be 10.7 ± 1.9 mm at 7 ± 2 months and 9.6 ± 1.4 mm at 18 ± 2 months for mean resorption of 19.5% and 27.8% at 7 months and 18 months, respectively. Histological examination demonstrated new bone and residual CO3Ap-granules of 33.8% ± 15.1% and 15.3% ± 11.9%, respectively, at 7 months (19). The rate of new bone formation from autogenous cortical bone alone is approximately 40%–50% (31, 32) and from bovine-derived xenograft is approximately 20%–30% (32–34). Hence, results from two-stage sinus floor augmentation compare positively with autogenous bone augmentation and bovine-derived xenograft only sinus augmentation. At 10 ± 2 months, sinus floor augmentation with CO3Ap-granules exhibited a reduction in elevated bone height (EBH) of 10.3% (19). This compares favorably with reduction in EBH for autogenous cortical bone augmentation of 14.4% in the iliac crest group at 5.5 months (35), reduction in EBH for autogenous bone in the mandible group of 8.4% at 5.5 months (35), and reduction in EBH for bovine-derived xenograft augmentation alone at 10 months of 6.5% (36).

A 3-year follow-up of the initial 2-stage sinus augmentation with CO3Ap-granules above was conducted with results revealing 100% implant survival without complications. Histological analyses at 7-month follow-up revealed mature new bone of 36.8% ± 17.3% and residual CO3Ap-granules of 16.2% ± 10.1% (37). These results compared quite favourably with a 9-month follow-up of bovine-derived xenograft with mature new bone of 19% and residual bovine-derived xenograft of 40% (38).

A clinical trial was conducted that compared CO3Ap-granules to deproteinized bovine-derived xenograft for sinus lift augmentation with 6 subjects receiving CO3Ap-granules sinus lift augmentation and 8 subjects receiving deproteinized bovine-derived xenograft sinus lift augmentation. At 6 months, the CO3Ap-granules group demonstrated significantly less bone resorption (*p *< 0.001) compared to the deproteinized bovine-derived xenograft group with 14.2% bone resorption for the CO3Ap-granules group and 24.2% bone resorption for the deproteinized bovine-derived xenograft group (39). Future clinical trials should be conducted to compare the relative efficacy of CO3Ap-granules and bovine-derived xenograft in other periodontal applications, such as immediate implant placement and as an adjunct to extraction site healing for eventual implant placement.

A clinical study was conducted to test the safety and efficacy of 0.3% basic fibroblast growth factor (REGROTH®, Kaken Pharmaceutical Co., Ltd., Tokyo, Japan) in combination with CO3Ap-granules in the treatment of severe alveolar bone defects as the result of moderate-to-severe periodontitis in 10 subjects. Currently, a flap operation (FO) in conjunction with fibroblast growth factor (FGF) has been the standard procedure for periodontal regenerative therapy in the treatment of alveolar bone defects in Japan. However, FGF has been inadequate in the treatment of severe alveolar bone defects. Hence, Kitamura, et al., conducted a clinical study of FGF and CO3Ap-granules to test the safety and efficacy of these two materials used together to treat severe alveolar bone defects using flap operations in these procedures. With the primary endpoint of safety, there were no adverse effects. In terms of secondary clinical endpoints at 36 weeks, significant increase in alveolar bone (*p* = 0.003), significant increase in CAL (*p* = 0.001), significant decrease in periodontal probing depth (*p* = 0.002), and significant decrease in bleeding on probing (*p* = 0.016) were observed compared to preoperative values. Future clinical studies comparing the combination of FGF and CO3Ap-granules to FGF alone and CO3Ap-granules alone need to be conducted to validate that the addition of CO3Ap-granules to FGF is advantageous in regenerating alveolar bone in the treatment of severe alveolar bone defects (40).

In a study published in 2023, a one-arm, single-center prospective trial was conducted to test the safety and efficacy of CO3Ap-granules in patients with periodontitis. Four patients with seven periodontitis sites were included in the study with the following sites included in the study: 3 deep intrabony defects, 2 class II furcation involvements, and 2 class III furcation involvements. At 9 months, reductions in probing depth were 5.0 ± 1.0 mm, 4.5 ± 0.7 mm, and 1.5 ± 0.7 mm for intrabony defects, class II furcation involvements, and class III furcation involvements, respectively. At 9 months, neither class III furcation involvements changed in terms of furcation classification. At 9 months, one class II furcation involvement improved to class I furcation involvement while the other class II furcation involvement was completely closed to eliminate furcation involvement (41).

Finally, the short-term histological results from three case reports for (1) an immediate implant placement, (2) a lateral guided bone regeneration (GBR), and (3) a vertical guided bone regeneration (GBR) demonstrated the efficacy of CO3Ap-granules in these three applications. In all three applications, new bone was produced while the original grafts of CO3Ap-granules were resorbed. In the limited results of these three short-term case reports, the novel alloplast CO3Ap-granules appears to be safe and effective. However, larger, long-term human clinical trials need to be conducted to validate results observed here and in the other human studies described previously (42).

### 3.4 Human case report of carbonate apatite crystals

**Figure 1:**
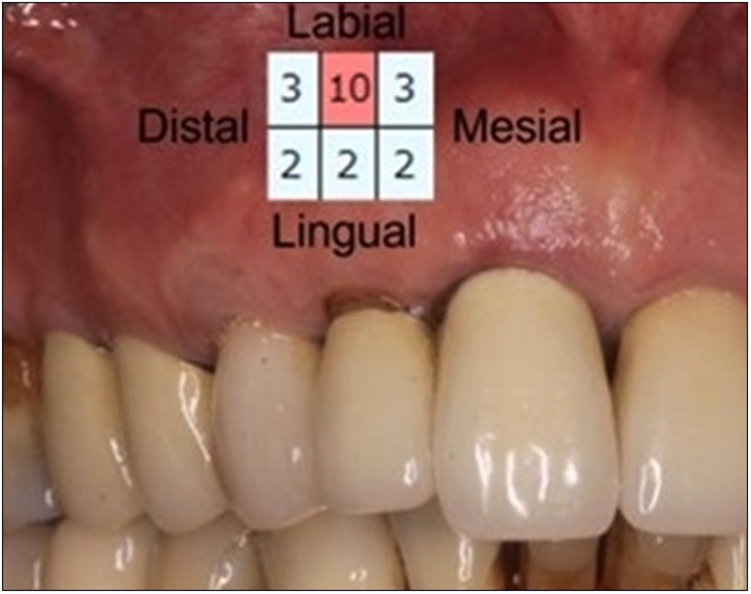
Clinical photograph of patient at regular supportive periodontal therapy appointment when 10 mm probing depth was detected. Insert shows recorded probing depths for all surfaces of #8.

**Figure 2:**
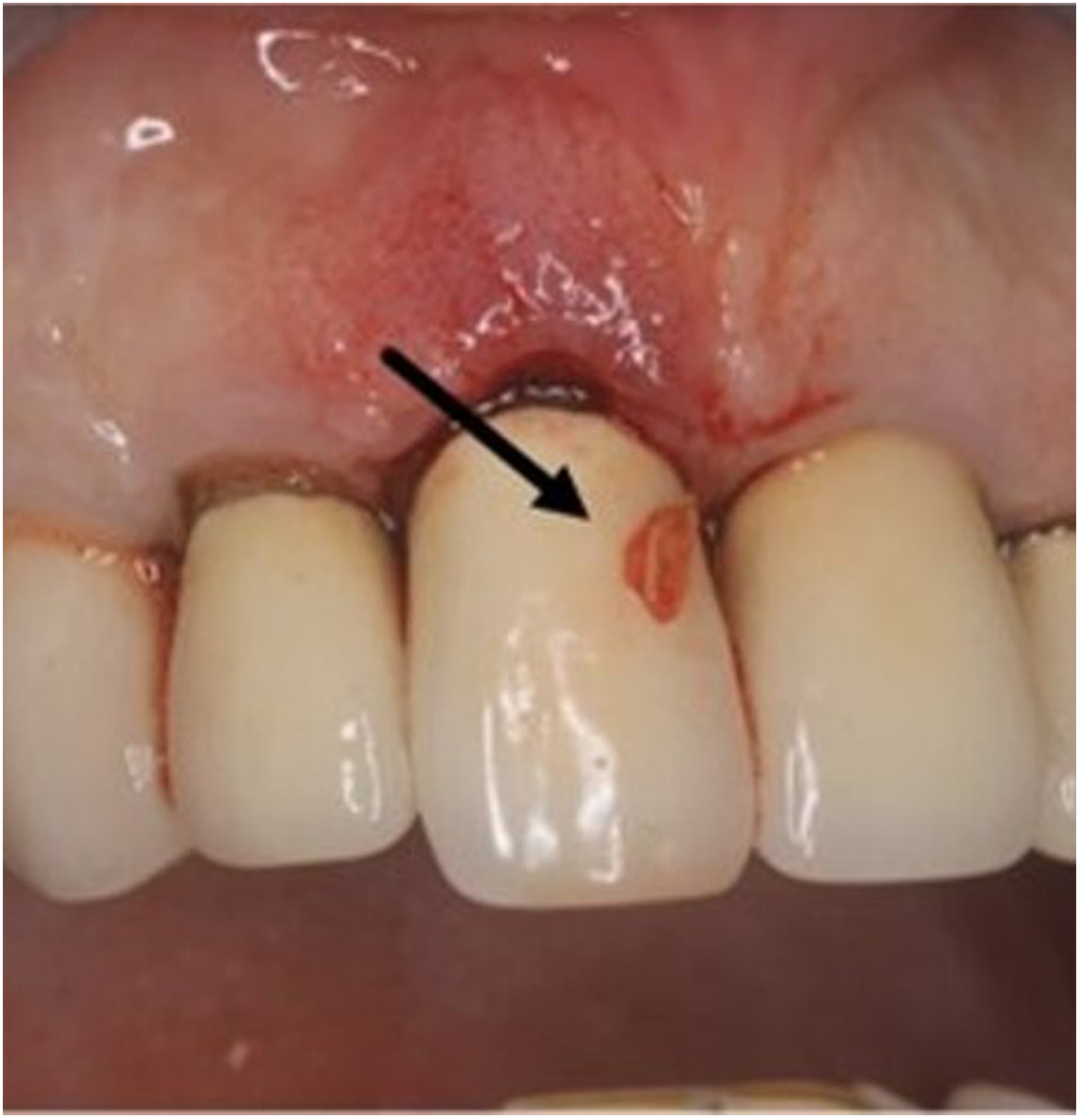
Clinical photograph of one of the cemental tear particles (black arrow) removed during ultrasonic scaling.

**Figure 3:**
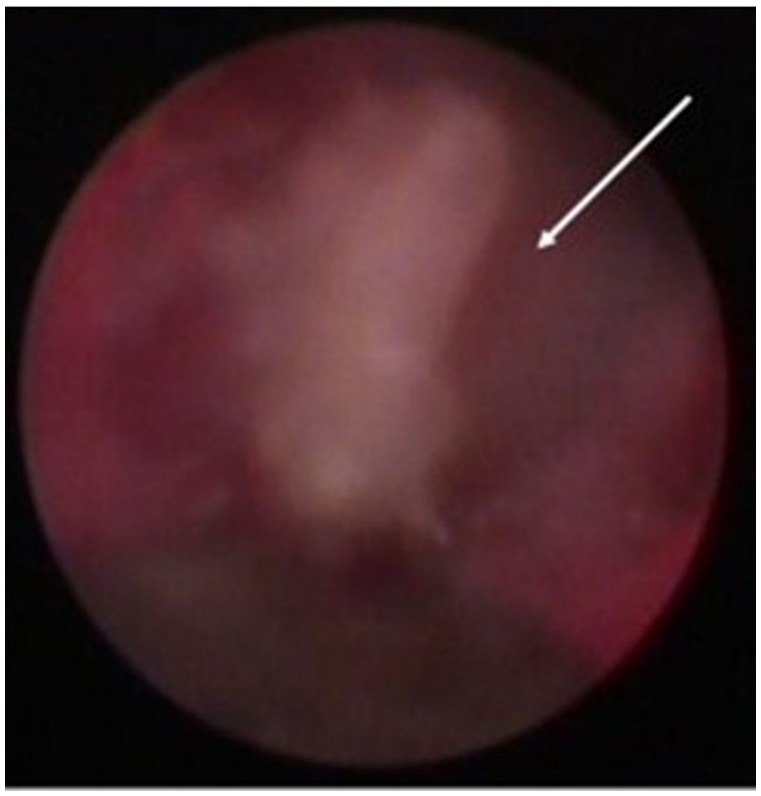
Screen grab from video taken with the periodontal endoscope showing a cemental tear particle (white arrow) embedded in the inner lining of the periodontal pocket causing inflammation and bone loss.

**Figure 4:**
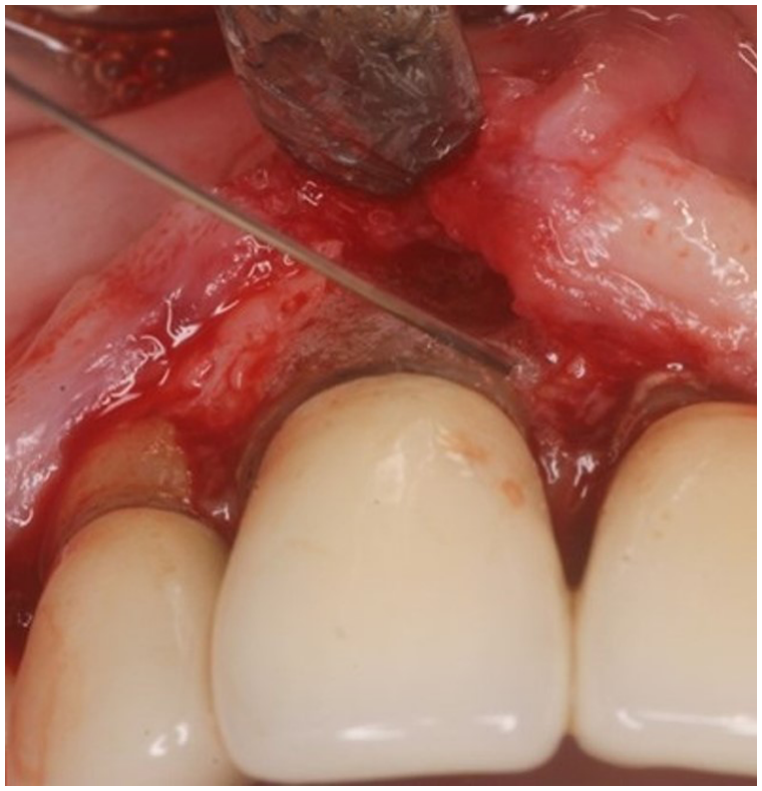
Clinical photograph showing vertical bone defect of #8 after flap resection and prior to debridement or grafting.

**Figure 5:**
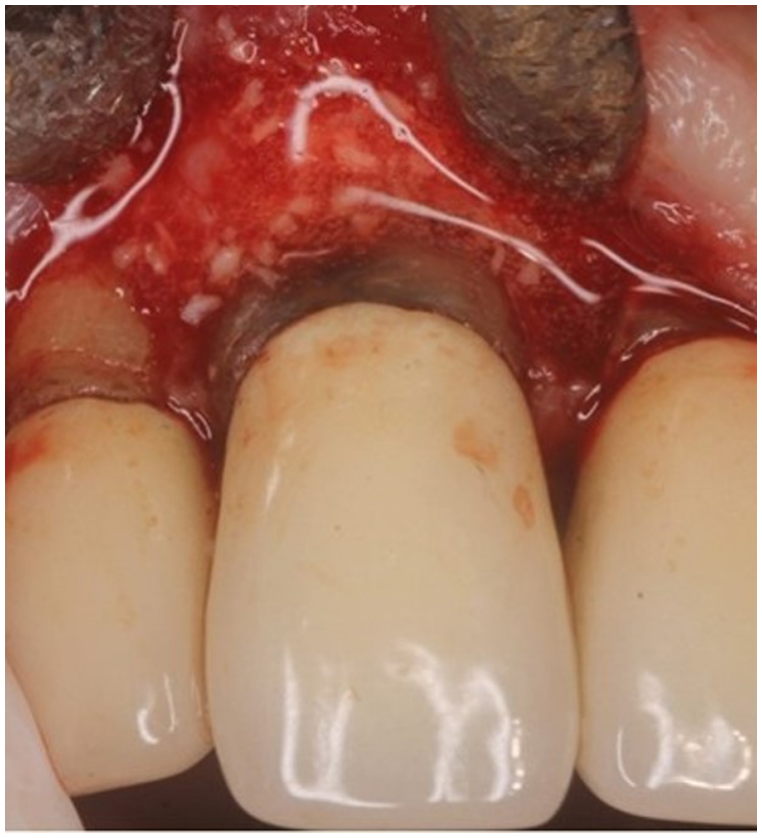
Carbonate apatite granules immediately after placement into debrided pocket.

**Figure 6:**
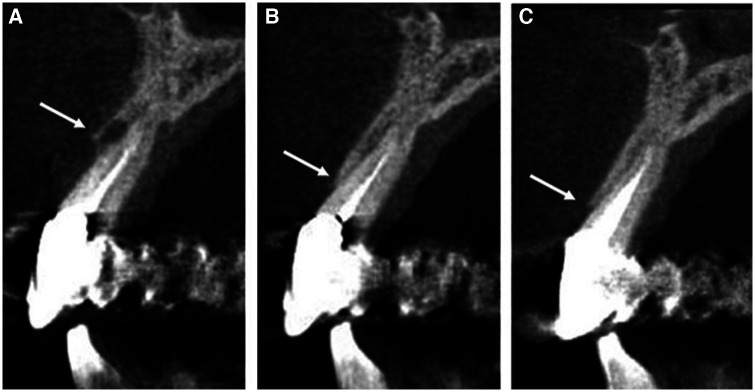
Computed tomography images showing bone levels (indicated by white arrows) at (**A**) initial presentation of pocketing during supportive periodontal therapy appointment with notable bone loss on the facial surface of #8, (**B**) 6-month follow up with visible bone regeneration and resorption of the carbonate apatite granules, (**C**) additional growth of regenerated bone at 18-month follow-up appointment.

In this case report, a 65-year-old male patient was seen by the same periodontal office (MN) over a 10-year period for supportive periodontal regenerative therapy. The patient was presented with a 10-mm periodontal probing depth localized to the facial of tooth number 8 – upper right central incisor (Figures 1–3). Initially, the etiology for this deep probing depth was thought to be a root fracture, which would have required the tooth to be extracted. However, the patient reported no other symptoms of a potential root fracture, so the area was examined using a periodontal endoscope. The periodontal endoscopy showed cemental tears resent with one large portion embedded in the facial gingiva adjacent to the bone defect (Figure 4). The separated cementum was removed using an ultra-sonic scaler (Figure 5). The area was then treated surgically to regenerate the alveolar bony defect. After flap elevation, additional cementum particles were removed, and the area was thoroughly debrided. FGF was applied over the root surface to expedite the keratinocyte epithelial-mesenchymal transition, and CO3Ap-granules were grafted into the area. In this case, CO3Ap-granules were held in place by the sutured gingiva with no additional material necessary for support or retention. At 6 months periodontal reevaluation visit, the periodontal probing depth had been reduced to 4 mm with no bleeding on probing. At the same appointment, a CT scan confirmed newly generated labial bone formation from the resorbed CO3Ap-granules at tooth number 8 (Figure 6). After 12 months, the periodontal probing depth remained stable, and a new zirconia crown was placed accordingly.

## 4 Discussion

The brief overview of existing hard-tissue grafting materials used in periodontics (shown in Supplementary Material), the systematic review of CO3Ap-granules, and the case report presented here demonstrate the potential advantages of the novel alloplastic hard-tissue grafting material, CO3Ap-granules, in periodontal regeneration. As pointed out in the brief overview of hard tissue grafting materials used in periodontics, autogenous bone grafts, previous allografts, and xenografts all have significant drawbacks when applied to periodontal surgery. Hence, a need for a safe, effective alloplastic osseous grafting material that can be applied to a wide variety of procedures within the field of periodontics has long existed (6, 8, 9).

Since the beginning of hard-tissue grafting in dentistry, clinicians and scientists have attempted to develop a hard-tissue grafting material with the most benefit and least risk to the patient. Currently, autografts are the ideal material because of its excellent osteoinductive and osteoconductive properties without the minimum risk of graft rejection. However, the primary drawbacks to autografts are limited source material, increased morbidity, and prolonged healing time for the patient from the harvesting site (6, 8, 9). The next best option to autografts would be an alloplastic material with the following properties: (1) has little to no risk of antigenicity or graft rejection, (2) can induce the generation of new bone, and (3) resorbs as new bone is formed. Previously, an alloplast with all these properties did not exist. Prior alloplasts failed to stimulate the formation of sufficient new bone while the alloplastic hard-tissue graft simultaneously remained in the grafting site to create problems (19). The lack of suitable alloplasts has left the dental profession with only two clinically viable options for hard-tissue grafts: xenografts and allografts, both of which have limitations. If an alloplastic material were developed which could induce both osteoclastic and osteoblastic function to promote the creation of new bone, could resorb over time as new bone is generated, and could promote angiogenesis to maintain the health and viability of the newly formed bone, the need for xenografts and allografts would virtually be eliminated, which, in turn, would drastically limit the risks to patients in need of alveolar bone regeneration.

Following Japan government approval in December, 2017 and market introduction in February, 2018, this novel alloplast, CO3Ap-granules, has been shown to be a consistently reliable hard-tissue grafting material in Japan with CO3Ap-granules becoming the most widely accepted dominant hard-tissue grafting material in Japan in 2022 (43). In addition, CO3Ap-granules received the prestigious “1st Japan Open Innovation Prize” in March, 2019, with only 12 out of 212 projects obtaining that recognition, which was established to promote and accelerate innovations in Japan (44). In August, 2020, the U.S. Food and Drug Administration (FDA) approved CO3Ap-granules as a hard-tissue grafting material with multiple dental applications (45). The official introduction of CO3Ap-granules to the United States market occurred in February, 2022 (43). The results of both animal and human studies demonstrate that CO3Ap-granules provide clinical results that are at least comparable to all other types of available hard-tissue grafting material (21, 23, 24). One of the clinical advantages of CO3Ap-granules is its ability to simultaneously resorb as new bone is deposited to replace the alloplastic hard-tissue graft. Both histologic studies and radiographic studies using CT radiography have demonstrated the utility of CO3Ap-granules in resorbing as new bone is acquired over time (19, 20).

There has long been a need for an effective, safe alloplastic osseous grafting material in periodontics. The applications for such an alloplast include repair of lost alveolar bone, elimination of periodontal pockets, clinical attachment regeneration, sinus floor augmentation, alveolar ridge augmentation, and as an adjunct in implant placement, particularly with sinus lifts, alveolar ridge augmentation, and to facilitate socket healing with placement of immediate implants. Previously, both xenografts, such as bovine-derived xenografts, and alloplasts, such as hydroxyapatite, have been limited in terms of resorption so that a high percentage of the original hard-tissue grafting material often remained in the surgical site. Based on both animal studies and clinical studies in humans, the amount of resorption of CO3Ap-granules and replacement with new bone is comparable to autogenous bone grafts with an average of 33.8% new bone at CO3Ap-granules sites at 7 months (19) compared to an average of 41.0% new bone at autogenous bone sites at 6 months (31) in human studies. In contrast, bovine-derived xenografts sites demonstrated an average of only 22.9% new bone at 8 months (34).

Taken together, the cumulative evidence provided by animal studies, human clinical studies, and the overwhelming acceptance of CO3Ap-granules in the Japanese dental market with steady growth in market share since 2018 demonstrates that CO3Ap-granules may be a hard-tissue grafting material worthy of clinical use and further clinical investigation (43). Overall, CO3Ap-granules can be promising allograft with multiple potential applications in periodontal surgery and oral surgery where a hard-tissue graft is potentially needed, including the repair of lost alveolar bone, periodontal alveolar reconstruction, gain in periodontal clinical attachment, alveolar ridge augmentation, as an adjunct in implant placement, and in the treatment of peri-implant disease.

The single greatest drawback of CO3Ap-granules is the lack of large randomized controlled clinical trials that would test the effectiveness of CO3Ap-granules compared to allografts, xenografts, and sham controls in a variety of periodontal hard-tissue grafting applications. It is suggested that future study is needed to demonstrate clinical efficacy and effectiveness of CO3Ap-granules through multi-center randomized controlled clinical trials.

## Data Availability

The original contributions presented in the study are included in the article/Supplementary Material, further inquiries can be directed to the corresponding author.
