# Supplementary material for: A systematic review of a novel alloplast carbonate apatite granules
Source: Front Dent Med. 2024 Aug 16;5:1418039. doi: 10.3389/fdmed.2024.1418039 (PMC11797801; doi:10.3389/fdmed.2024.1418039)
Supplement: Supplementary file 1 [file Datasheet1.docx]

**Brief Overview and Comparison of Hard Tissue Grafts used in Periodontics**

*Autogenous Bone Graft*

Autogenous bone grafting from an intraoral source to repair osseous defects as a result of periodontal disease was first attempted by Hegedus in 1923 [1,2]. Autogenous bone grafting was revived by Nabers and O’Leary in 1965 by utilizing cortical bone shavings applied to the surgical site [3]. The resulting autogenous bone graft provided an increase in bone height coronally [4].

Another type of autogenous bone graft described by Robinson in 1969 involves a mixture of bone powder and blood referred to as osseous coagulum. Two major disadvantages to osseous coagulum were the inability to use aspiration while placing the mixture and the questionable quality and quantity of bone particles in the mixture [5]. Other intraoral autogenous bone grafts include: (1) a bone blend technique where a combination of cortical and cancellous bone is triturated into a workable mass that can be packed into osseous defects, (2) a bone swaging technique where bone from an edentulous area adjacent to the osseous defect is pushed into contact with the root surface while keeping the bone at the base of the edentulous area intact, and (3) cancellous bone obtained from healing sockets, edentulous areas, and maxillary tuberosity [2].

Between 1968 and 1972, Schallhorn, et al., used iliac bone marrow biopsy and iliac cancellous bone for treatment of furcation and osseous defects and were able to gain as much as 3 to 4 mm in crestal bone [6,7,8]. In addition to the added risk, time, and expense associated with the surgical procedure required to obtain iliac autogenous grafting material, there is also the problem that grafting material obtained from the iliac contains the monoblastic precursor to osteoclasts so that resorption may be observed when the graft is placed adjacent to a tooth root [4]. Autogenous bone grafts are the gold standard for hard-tissue grafts because of its osteogenic, osteoconductive, and osteoinductive properties, as well as no risk of host rejection, infection transmission, and minimal inflammatory response [4]. However, the risk of infection, inflammation, and ongoing pain at the donor site presents a problem, whether the source is intraoral or extraoral. A systematic review and meta-analysis was conducted by McKenna, et al., to compare iliac autogenous grafts to intraoral autogenous grafts on implant survival. Out of the 23 studies that were included in the systematic review, 14 provided implant survival data. Based on implant survival, significantly longer implant survival was observed for implants with intraoral autogenous grafts compared to implants with iliac autogenous grafts. Significantly better survival for implants placed with intraoral autogenous grafts compared to implants placed with iliac autogenous grafts was observed for 6 months, 12 months, 24 months, 60 months, and 120 months. At 6 months, dental implants placed with intraoral autogenous bone grafts had a 98.4% survival rate compared to 95.8% survival rate for dental implants placed with iliac autogenous bone grafts. At 120 months, dental implants placed with intraoral autogenous bone grafts had a 95.2% survival rate compared to 88.8% survival rate for dental implants placed with iliac autogenous bone grafts [9].

*Allograft*

An allograft is bone from a genetically dissimilar individual of the same species. The two most common types of bone allografts used in periodontics are (1) Freeze-Dried Bone Allograft (FDBA) and (2) Demineralized Freeze-Dried Bone Allograft (DFDBA). Mellonig, et al., published some of the early studies of allograft (FDBA and DFDBA) applied to periodontics [10,11]. Initial studies by Mellonig, et al, involved FDBA with later studies including DFDBA with significantly better results obtained from DFDBA compared to FDBA [12]. In another study by Sanders, et al., osseous defects were randomly treated with either FDBA or a composite of FDBA and autogenous bone with the composite of FDBA and autogenous bone providing significantly better results [13]. In a systematic review of bone grafting materials, autogenous bone grafts were found to have significantly more vital bone and less soft connective tissue compared to allogenic bone grafts [14].

Allografts are the most frequent alternative to autogenous bone for bone grafting procedures in periodontics in the United States [15]. A DFDBA has an osteoconductive surface and osteoinductive factors. For FDBA and DFDBA, the freeze-drying process drastically reduces the risk of disease transmission and antigenicity [2].

*Xenograft*

A xenograft is bone obtained from a different species than humans. Three sources of xenografts used in periodontics are: (1) bovine-derived bone xenograft, (2) natural coral, and (3) porcine-derived bone and collagen xenografts. Bovine bone and natural coral are structurally similar to human bone and are biocompatible to human bone [16].

Bovine-derived xenograft consists of deproteinized, sterile bovine bone with 75-80% porosity and a crystal size of approximately 10 mm in the form of cortical granules. Examples of bovine-derived xenograft include Bio-Oss^®^ (Osteohealth Co., Shirley, NY) and Osteograf/N^®^ (CeraMed Dental, LLC, Lakewood, CO). The advantages of bovine-derived xenografts are the following: (1) it is easily manipulated and (2) it has predictably positive results when proper surgical technique is utilized [17,18]. Since some European countries restrict the importation of human-derived hard tissue grafting material, this is another advantage of xenografts. The disadvantages include the following: (1) the potential for antigenicity, and (2) possible disease transmission, such as bovine spongiform encephalopathy in the United Kingdom (i.e., “mad cow disease”), although the risk of disease transmission from xenografts is relatively low [19].

Porcine-derived collagen is obtained from healthy pigs [2]. Porcine-derived hard tissue xenografts with a particle size of 250-1000 µm are available as MinerOss XP^®^ (Biohorizons, Birmingham, AL). Porcine-derived hard tissue xenografts and porcine-derived collagen xenografts resulted in less ridge resorption following extraction compared to extraction sites not treated with porcine-derived bone or porcine-derived collagen xenografts [17]. A randomized controlled clinical trial was conducted to compare the histological and dimensional properties of ridge preservation with porcine-derived xenografts versus bovine-derived xenografts. Porcine-derived xenografts resulted in similar dimensional stability and histological properties as bovine-derived xenografts. While no statistically significant differences between the porcine-derived xenograft group and bovine-derived xenograft group were detected in terms of new bone formation, more implants in the porcine-derived xenograft group required additional hard tissue grafts at the time of implant placement [20].

Coralline calcium carbonate is a natural coral xenograft derived from the exoskeleton of coral and marketed as Biocoral^®^ (Inoteb, Saint Gonnery, France). In a clinical trial that compared bovine-derived xenograft, coral-derived xenograft, and a synthetic sponge graft based on polylactic-polyglycolic acid technology was conducted. The synthetic sponge graft had significantly less residual biomaterial at 2 months at 5.6%±8.9% compared to the coral-derived xenograft with 12%±16.4% and the bovine-derived xenograft with 20.2%±17%. All grafts provided primary stability and rigidity for implant placement [21].

*Alloplast*

An alloplast is a completely synthetic or inorganic hard-tissue grafting material. Alloplasts are classified by ability to be bioabsorbable. Bioabsorbable materials include ceramics, β‑tricalcium phosphate, hydroxyapatite (HA), carbonate apatite (CA), calcium sulfate (CS), and calcium carbonate (CC). Non-bioabsorbable materials include porous hydroxyapatite (porous HA), dense hydroxyapatite (dense HA), bioglass (BG), and a calcium‑coated polymer of hydroxyl ethyl methacrylate and polymethyl methacrylate (PMMA). Since alloplasts are synthetic, risk of antigenicity and disease transmission is virtually eliminated. In addition, alloplasts are biocompatible, long-term resorbable with replacement by host bone, available in particulate and molded forms, easy to manipulate, no growth of oral pathogens, nonallergenic, and positive clinical results, such as reduction in probing depths, increased clinical attachment levels, and hard tissue fill of intra-bony defects. The disadvantages of alloplasts include longer recovery time, variability in repair, unpredictable outcome, and greater expense [2].

**References**

1. Hegedus Z. The rebuilding of the alveolar process by bone transplantation. *Dental Cosmos* **1923**, 736.
2. Balaji VR, Manikandan D, Ramsundar A. Bone grafts in periodontics. *Matrix Science Medica* **2020**, 4, 3, 57-63; DOI:10.4103/MTSM.MTSM_2_19.
3. Nabers CL, O'Leary TJ. Autogenous bone transplants in the treatment of osseous defects. *Journal of Periodontology* **1965**, 36, 5-14; DOI:10.1902/jop.1965.36.1.5.
4. Kumar J, Jain V, Kishore S, Pal H. Journey of bone graft materials in periodontal therapy: A chronological review. *Journal of Dental and Allied Sciences* **2016**, 5, 1, 30; DOI: 10.4103/2277-4696.185195.
5. Robinson RE. Osseous coagulum for bone induction. *Journal – California Dental Association* **1970**, 46, 1, 18-27; PMID: 4911837.
6. Schallhorn RG. The use of autogenous hip marrow biopsy implants for bony crater defects. *Journal of Periodontology* **1968**, 39, 3, 145-147; DOI:10.1902/jop.1968.39.1.145.
7. Schallhorn RG, Hiatt WH, Boyce W. Iliac transplants in periodontal therapy. *Journal of Periodontology* **1970**, 41, 10, 566-580; DOI:10.1902/jop.1970.41.41.566.
8. Schallhorn RG, Hiatt WH. Human allografts of iliac cancellous bone and marrow in periodontal osseous defects. II. Clinical observations. *Journal of Periodontology* **1972**, 43, 2, 67-81; DOI:10.1902/jop.1972.43.2.67.
9. McKenna GJ, Gjengedal H, Harkin J, Holland N, Moore C, Srinivasan M. Effect of autogenous bone graft site on dental implant survival and donor site complications: A systematic review and meta-analysis. *Journal of Evidence-Based Dental Practice* **2022**, 22, 3, 101731; DOI:10.1016/j.jebdp.2022.101731.
10. Mellonig JT, Bowers GM, Bright RW, Lawrence JJ. Clinical evaluation of freeze-dried bone allografts in periodontal osseous defects. *Journal of Periodontology* **1976**, 47, 3, 125-131; DOI:10.1902/jop.1976.47.3.125.
11. Quintero G, Mellonig JT, Gambill VM, Pelleu GB Jr. A six-month clinical evaluation of decalcified freeze-dried bone allografts in periodontal osseous defects. *Journal of Periodontology* **1982**, 53, 12, 726-730; DOI:10.1902/jop.1982.53.12.726.
12. Mellonig JT. Decalcified freeze-dried bone allograft as an implant material in human periodontal defects. *International Journal of Periodontics and Restorative Dentistry* **1984**, 4, 6, 40-55; PMID: 6396269.
13. Sanders JJ, Sepe WW, Bowers GM, Koch RW, Williams JE, Lekas JS, Mellonig JT, Pelleu GB Jr, Gambill V. Clinical evaluation of freeze-dried bone allografts in periodontal osseous defects. Part III. Composite freeze-dried bone allografts with and without autogenous bone grafts. *Journal of Periodontology* **1983**, 54, 1, 1-8; DOI: 10.1902/jop.1983.54.1.1.
14. Spin-Neto R, Stavropoulos A, Coletti FL, Faeda RS, Pereira LA, Marcantonio E Jr. Graft incorporation and implant osseointegration following the use of autologous and fresh-frozen allogeneic block bone grafts for lateral ridge augmentation. *Clinical Oral Implants Research* **2014**, 25, 2, 226-233; DOI:10.1111/clr.12107.
15. Reynolds MA, Aichelmann-Reidy ME, Branch-Mays GL. Regeneration of periodontal tissue: bone replacement grafts. *Dental Clinics of North America* **2010**, 54, 1, 55-71; DOI:10.1016/j.cden.2009.09.003.
16. Sukumar S, Drízhal I. Bone grafts in periodontal therapy. *Acta Medica (Hradec Kralove)* **2008**, 51, 4, 203-107; PMID: 19453085.
17. Guarnieri R, Testarelli L, Stefanelli L, De Angelis F, Mencio F, Pompa G, Di Carlo S. Bone Healing in Extraction Sockets Covered With Collagen Membrane Alone or Associated With Porcine-Derived Bone Graft: a Comparative Histological and Histomorphometric Analysis. *Journal of Oral and Maxillofacial Research* **2017**, 8, 4, e4; DOI: 10.5037/jomr.2017.8404.
18. Cornelius Timothius CJ, Kilic HN, Gandi KK, Kakar A, John V. Particulate bone graft materials for periodontal and implant surgery: A narrative review and case series. *Dentistry Review* **2023**, 3, 2, 100068; DOI:10.1016/j.dentre.2023.100068.
19. Piattelli M, Favero GA, Scarano A, Orsini G, Piattelli A. "Bone reactions to anorganic bovine bone (Bio-Oss) used in sinus augmentation procedures: a histologic long-term report of 20 cases in humans." *International Journal of Oral and Mxillofacial Implants* **1999**, 14, 6, 835-840; PMID: 10612920.
20. Lai VJ, Michalek JE, Liu Q, Mealey BL. Ridge preservation following tooth extraction using bovine xenograft compared with porcine xenograft: A randomized controlled clinical trial. *Journal of Periodontology* **2020**, 91, 3, 361-368; DOI:10.1002/JPER.19-0211.
21. Molly L, Vandromme H, Quirynen M, Schepers E, Adams JL, van Steenberghe D. Bone formation following implantation of bone biomaterials into extraction sites. *Journal of Periodontology* **2008**, 79, 6, 1108-1115; DOI:10.1902/jop.2008.070476.
