# Supplementary figures and images for: A systematic review of a novel alloplast carbonate apatite granules
Source: Front Dent Med. 2024 Aug 16;5:1418039. doi: 10.3389/fdmed.2024.1418039 (PMC11797801; doi:10.3389/fdmed.2024.1418039)

Diagram 1: PRISMA Flowchart for systematic review of carbonate apatite

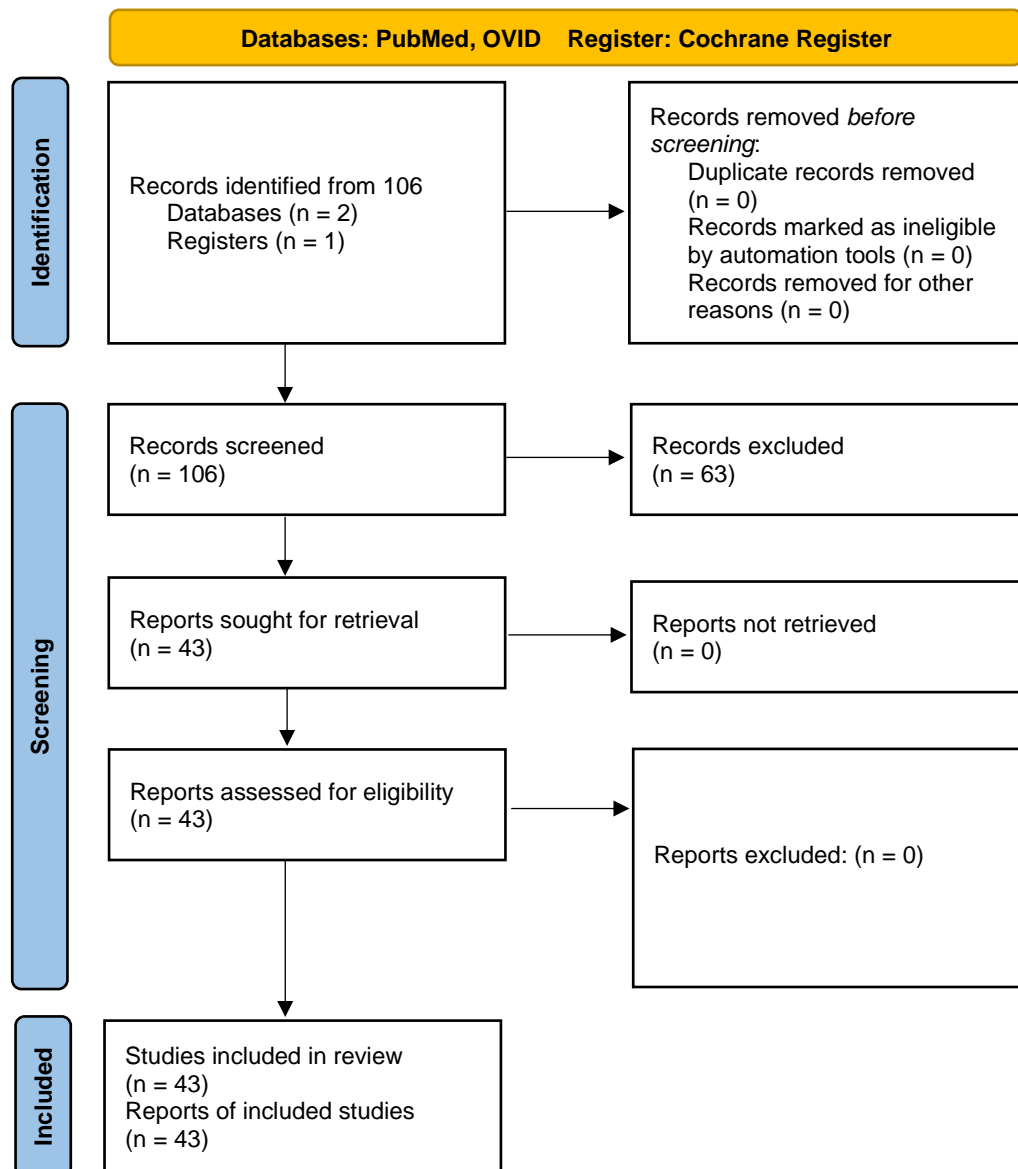

Supplement: Supplementary file 3 [file Image1.pdf]
